# Biohydrogen production of obligate anaerobic archaeon *Thermococcus onnurineus* NA1 under oxic conditions via overexpression of *frhAGB*-encoding hydrogenase genes

**DOI:** 10.1186/s13068-019-1365-3

**Published:** 2019-02-08

**Authors:** Seong Hyuk Lee, Min-Sik Kim, Sung Gyun Kang, Hyun Sook Lee

**Affiliations:** 1 Korea Institute of Ocean Science and Technology, Busan, 49111 Republic of Korea; 2 Biomass and Waste Energy Laboratory, Korea Institute of Energy Research, Daejeon, 34129 Republic of Korea; 3 Department of Marine Biotechnology, Korea University of Science and Technology, Daejeon, Republic of Korea

**Keywords:** *frhAGB*-encoding hydrogenase, Obligate anaerobe, *Thermococcus onnurineus* NA1, O_2_ tolerance, Biohydrogen

## Abstract

**Background:**

The production of biohydrogen (H_2_) as a promising future fuel in anaerobic hyperthermophiles has attracted great attention because H_2_ formation is more thermodynamically feasible at elevated temperatures and fewer undesired side products are produced. However, these microbes require anoxic culture conditions for growth and H_2_ production, thereby necessitating costly and time-consuming physical or chemical methods to remove molecular oxygen (O_2_). Therefore, the development of an O_2_-tolerant strain would be useful for industrial applications.

**Results:**

In this study, we found that the overexpression of *frhAGB*-encoding hydrogenase genes in *Thermococcus onnurineus* NA1, an obligate anaerobic archaeon and robust H_2_ producer, enhanced O_2_ tolerance. When the recombinant FO strain was exposed to levels of O_2_ up to 20% in the headspace of a sealed bottle, it showed significant growth. Whole transcriptome analysis of the FO strain revealed that several genes involved in the stress response such as chaperonin β subunit, universal stress protein, peroxiredoxin, and alkyl hydroperoxide reductase subunit C, were significantly up-regulated. The O_2_ tolerance of the FO strain enabled it to grow on formate and produce H_2_ under oxic conditions, where prior O_2_-removing steps were omitted, such as the addition of reducing agent Na_2_S, autoclaving, and inert gas purging.

**Conclusions:**

Via the overexpression of *frhAGB* genes, the obligate anaerobic archaeon *T. onnurineus* NA1 gained the ability to overcome the inhibitory effect of O_2_. This O_2_-tolerant property of the strain may provide another advantage to this hyperthermophilic archaeon as a platform for biofuel H_2_ production.

**Electronic supplementary material:**

The online version of this article (10.1186/s13068-019-1365-3) contains supplementary material, which is available to authorized users.

## Background

Anaerobic microbes play important roles in a variety of biotechnological processes such as fermented food production, biochemical synthesis, biofuel production, and bioremediation. For the cultivation and manipulation of these microbes, however, specialized methods are required to maintain anoxic culture conditions. O_2_ is potentially toxic to anaerobes; however, anaerobes also have mechanisms to cope with toxic oxygen species such as superoxide anions (O_2_^−^), hydrogen peroxide (H_2_O_2_), and free hydroxyl radicals (OH•) [1, 2]. To create an O_2_-free environment and the low redox potential that is essential for anaerobic growth, numerous methods have been employed [3]. For instance, deaeration of nutrient medium by boiling is the simplest way to drive absorbed O_2_ out of a culture medium by reducing the solubility of gases at the temperature of boiling water. The combination of evacuation and purging of vials with O_2_-free gas facilitates the reduction of O_2_ tension. Chemical reducing agents containing sulfur, such as cysteine hydrochloride (*E*_0_′ = − 210 mV, redox potential defined at pH 7 and 298 K), sodium thioglycollate (*E*_0_′ = − 140 mV), or Na_2_S (*E*_0_′ = − 571 mV), are very effective at maintaining the low redox potential of the medium [4, 5]. Even though various physical or chemical methods are effective at keeping the culture medium anaerobic, these strategies are costly and time consuming.

Hyperthermophiles have advantages in biotechnological applications because of reduced burden on reactor cooling processes, low chance of contamination, and increased reaction rates [6]. Biohydrogen (H_2_) production using hyperthermophiles is furthermore advantageous because, at elevated temperatures, H_2_ formation is more thermodynamically feasible and fewer undesired side products are produced [7, 8]. *Thermococcus onnurineus* NA1 is a hyperthermophilic obligate anaerobic archaeon that is capable of producing H_2_ using starch, formate, or carbon monoxide (CO). It has been recently reported that *T. onnurineus* NA1 possesses high H_2_ production rates during growth on formate, comparable to those of various bacteria and archaea with a formate dehydrogenase and a hydrogenase in the form of formate hydrogen lyase (FHL) or hydrogen-dependent CO_2_ reductase (HDCR), or separately [9–17]. In particular, H_2_ production by *T. onnurineus* NA1 using steel-mill waste gas was successfully demonstrated, indicating that environmentally friendly H_2_ production is feasible [18, 19]. Over the years, H_2_ production by this strain has been improved by employing various strategies of genetic engineering [18, 20, 21], adaptive laboratory evolution [22, 23], and fermentation process engineering [24]. Even though the strain has great potential for practical applications as an H_2_ producer, it must be carefully handled and cultivated to prevent exposure to O_2_ in all the steps. In addition to inhibition of cell growth, H_2_ production is also inhibited by O_2_ since membrane-bound [NiFe] hydrogenases, involved in H_2_ evolution, are O_2_ sensitive to some degree [25, 26].

In this study, we present a recombinant strain of *T. onnurineus* NA1 that can grow and produce H_2_ under oxic conditions, where any physical or chemical methods were not applied to remove O_2_ from the medium and the bioreactor headspace, which is a condition under which the wild-type strain cannot grow at all. The FO strain exhibited a very similar cell yield and only a 10% reduction in the H_2_ production rate compared to the strain grown under anoxic conditions. This study may enhance the prospects of exploiting this obligate anaerobe as a robust tool for biotechnology.

## Results

### Construction and phenotype of a recombinant strain (FO)

**Fig. 1:**
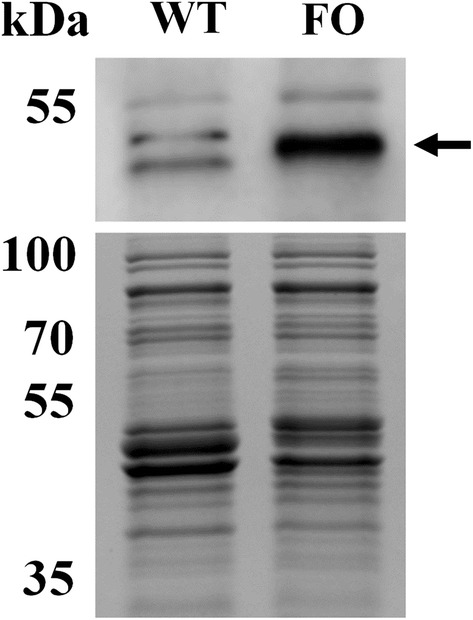
Expression of the *frhA* gene in *T. onnurineus* NA1. Western blot analysis of the expression of *frhA*-encoding protein in the FO strain (upper panel). The position of the target protein is denoted by an arrow. SDS-PAGE gel was stained with Coomassie Brilliant Blue (lower panel). *WT* wild-type strain, *FO* a recombinant strain overexpressing *frhAGB*; the numbers denote molecular masses in kilodaltons

**Fig. 2:**
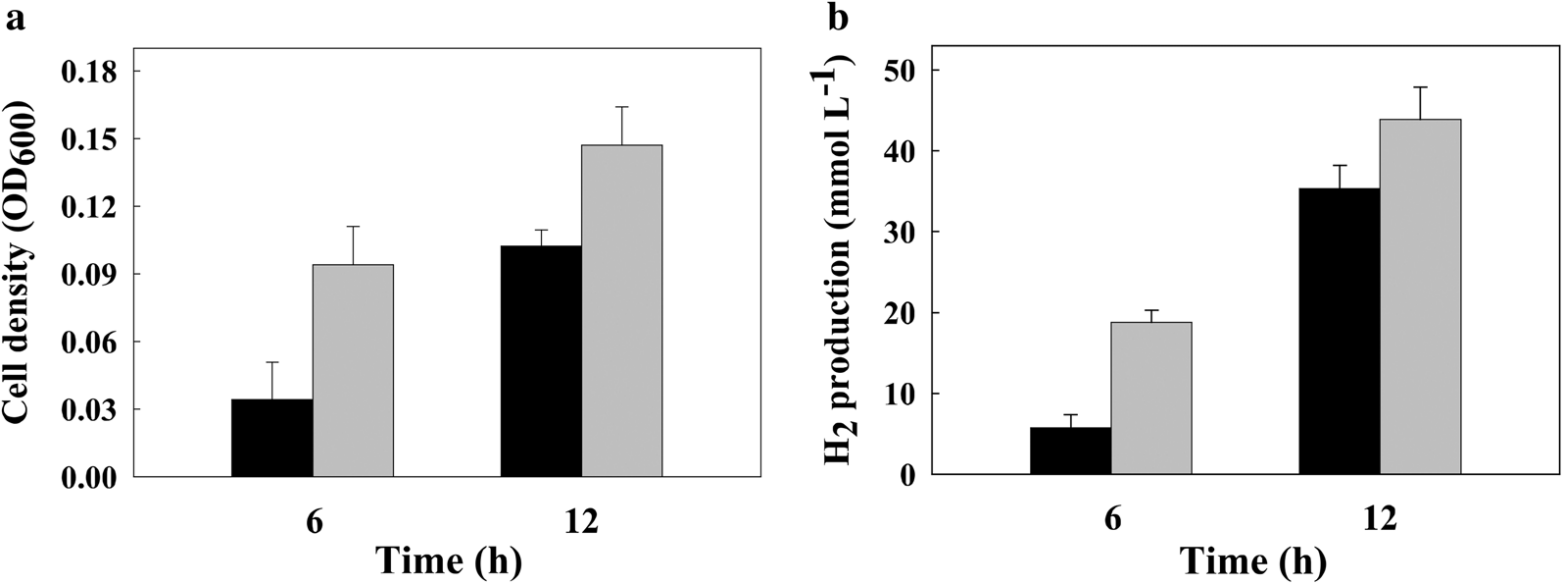
Growth and H_2_ production of the wild-type and FO strains in formate-supplemented medium. Growth (**a**) and H_2_ concentration (**b**) of the wild-type (black bars) and FO (gray bars) strains were monitored at the indicated time points. Cell growth was monitored by measuring the optical density at 600 nm (OD_600_). All experiments were carried out twice independently, each in triplicate

In our previous report, the *frhAGB*-encoding hydrogenase from *T. onnurineus* NA1, homologous to the F_420_-reducing hydrogenases, a key enzyme in methanogenesis, was characterized [27]. To obtain a higher yield of the enzyme complex for biochemical studies, the *frhABG* operon was overexpressed in the native strain using a strong constitutive promoter, resulting in the FO strain (Additional file 1: Fig. S1) [27]. The production of *frhAGB* in the FO strain was measured by Western blot, and the Frh α subunit encoded by the *frhA* gene was observed to be markedly increased (Fig. 1). The FO strain grew well using formate as an energy source and showed 3.4- and 1.4-fold higher cell densities at 6 and 12 h of culture, respectively, than those of the wild-type strain (Fig. 2a). The *fdh2*–*mfh2*–*mnh2* gene cluster was previously determined to be essential for the oxidation of formate and subsequent production of H_2_ coupled with ATP synthesis [10]; therefore, the concentration of H_2_ produced as a product of formate oxidation was measured. The FO strain produced 2.8- and 1.4-fold higher amounts of H_2_ at the two measured time points than those of the wild-type strain, respectively, which correspond to the increasing levels of cell density (Fig. 2b). These results indicated that the expression level of membrane-bound formate-dependent hydrogenase (Mfh2) was not changed, and the protein level of a large subunit of Mfh2 hydrogenase was confirmed to be similar to that of the wild-type strain (Additional file 1: Fig. S2).

### Transcriptome analysis of the FO strain

To explore the physiological changes that occurred through the overexpression of *frhAGB* genes, the expression changes were investigated at the whole transcriptome level. We performed microarray-based transcriptome analysis using samples of the wild-type and FO strains that were cultured in formate-supplemented medium under the anoxic condition. The results showed that 24 and 33 genes were identified as being up- and down-regulated with fold changes of ≥ 2 or ≤ 0.5 in expression compared with the wild-type strain, respectively (Additional file 1: Tables S1, S2). Among the down-regulated group, a gene cluster was identified to be related to the biosynthesis of disaccharides, oligosaccharides and polysaccharides (locus tag TON_1857 ~ 1862), including galactosyltransferases and glycosyltransferases. Some of the genes encoding proteins such as sulfate adenylyltransferase (TON_1707), transcriptional regulatory protein (TON_0836 and TON_1663), and ATP-dependent helicase (TON_1042 and TON_1380) were down-regulated. Among the up-regulated group, three gene clusters were identified, whose functions are predicted as transport (TON_0014 ~ 0015 and TON_0656 ~ 0657) and thiamine biosynthesis (TON_0853 ~ 0854). Two genes related to the stress response, chaperonin β subunit (TON_1877) and universal stress protein (TON_1493), were also up-regulated. Interestingly, two genes encoding peroxiredoxin (TON_0786) and alkyl hydroperoxide reductase subunit c (TON_0847), which are known to protect cells against oxidative stress by reducing hydrogen peroxide and/or alkyl hydroperoxides, were up-regulated.

### O_2_ tolerance test

**Fig. 3:**
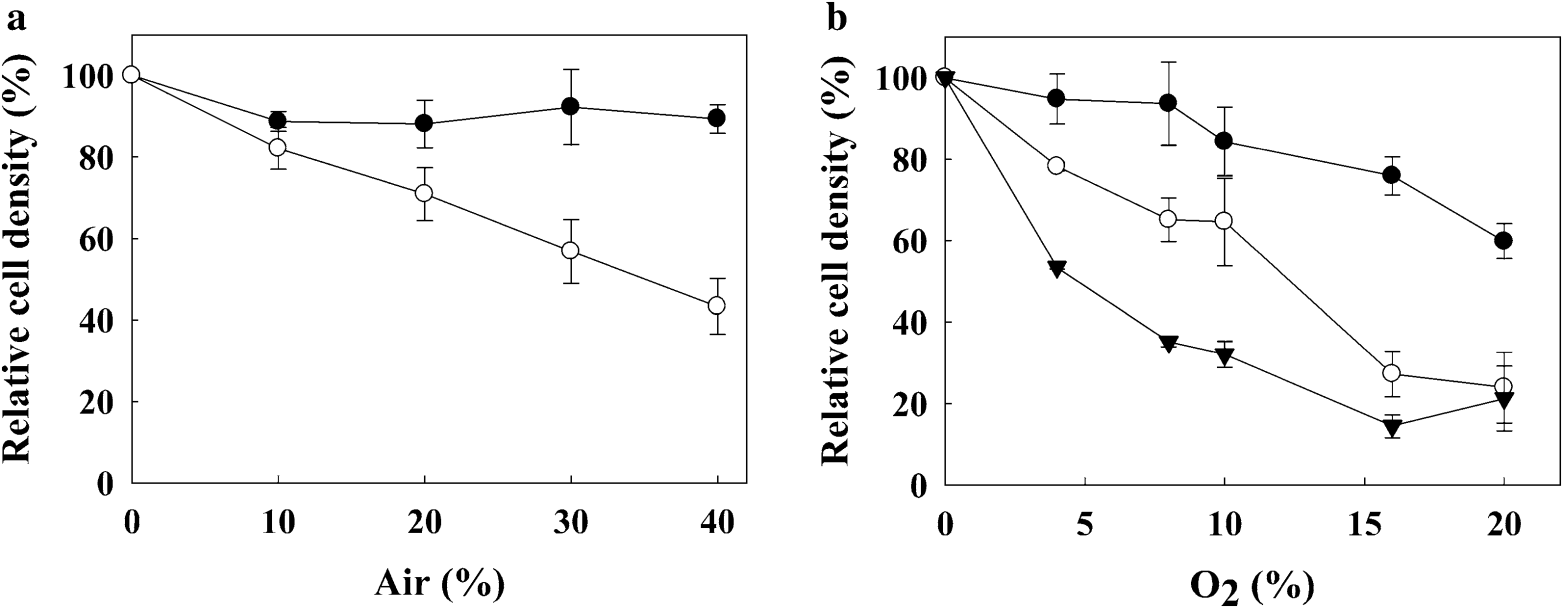
O_2_ sensitivity at various concentrations of air or O_2_. Wild-type (open circles), FO (closed circles), and Δ*frhA* mutant (closed inverted triangles) strains were cultured in formate-supplemented medium in the presence of 0, 10, 20, 30, 40% (v/v) of air (**a**) or 0, 4, 8, 10, 16, or 20% (v/v) of O_2_ (**b**) in the headspace. Cell densities of the wild-type strain cultured in the presence of air and O_2_ were 0.09 ± 0.02 and 0.09 ± 0.03, respectively, and each was set to 100%. Cell densities of the FO strain cultured in the presence of air and O_2_ were 0.14 ± 0.03 and 0.118 ± 0.006, respectively, and each was set to 100%. Cell density of the Δ*frhA* mutant strain cultured in the presence of O_2_ was 0.069 ± 0.007 and was set to 100%. Cell growth was monitored by measuring the optical density at 600 nm (OD_600_) after 12-h cultivation. All experiments were carried out twice independently, each in triplicate

Based on the transcriptomic data, we speculated that the changes at the transcriptional level of several stress response genes caused by *frhAGB* overexpression might affect O_2_ tolerance of the strain. Therefore, we compared O_2_ sensitivity between the wild-type and FO strains. Each strain was cultured in sealed vials containing 0, 10, 20, 30, and 40% of atmospheric air (21% O_2_, v/v) in the headspace. With increasing percentages of atmospheric air, the wild-type strain showed gradual decreases in cell density, ranging from 17.9 to 56.7% of the control (0% air) (Fig. 3a). On the other hand, the growth of the FO strain remained constant, with changes in cell density ranging from only 7.7 to 11.9%, indicating a significant enhancement in O_2_ tolerance.

In our previous report, the *frhA*-deletion mutant, Δ*frhA*, showed very limited changes in cell density and H_2_ production compared with the wild-type strain [28]. To correlate the intracellular level of the *frhAGB*-encoding hydrogenase with O_2_ tolerance, we tested how the O_2_ sensitivities of the wild-type strain, the *frhA*-deletion mutant strain, and the FO strain were affected at higher concentrations of O_2_ in the headspace of culture vials. Instead of air, pure O_2_ was added to a concentration of 20%, equivalent to the atmospheric concentration of O_2_. Once again, the FO strain grew much better than the wild-type strain (Fig. 3b). On the other hand, the Δ*frhA* mutant strain was more sensitive to O_2_ than the wild-type strain.

### Growth of the FO strain under oxic conditions

**Fig. 4:**
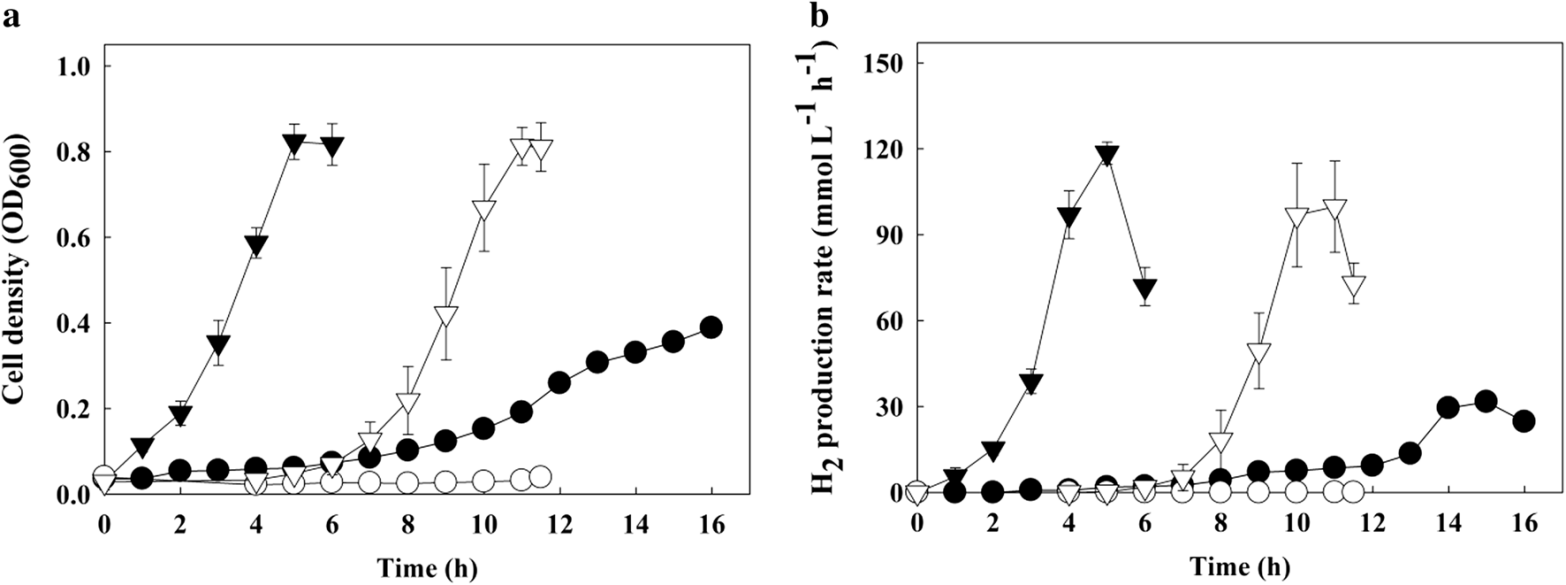
Growth (**a**) and H_2_ production (**b**) of the FO strain in a bioreactor under oxic conditions omitting the addition of reducing agent Na_2_S, autoclaving, and inert gas purging. The wild-type (circles) and FO (inverted triangles) strains were cultured under oxic (open symbols) or anoxic (closed symbols) conditions in a 3-L bioreactor with 4 g L^−1^ of yeast extract and 400 mM sodium formate as a substrate. Cell growth was monitored by measuring the optical density at 600 nm (OD_600_). pH was adjusted to 6.1–6.2 using 2 N HCl containing 3.5% NaCl or 0.5 M citric acid containing 4% NaCl. Error bars indicate standard deviation of three independent cultures of the FO strain, and the data for the wild-type strain under anoxic conditions were adapted from previously reported data [23]

Since the FO strain showed strong performance in the presence of O_2_, we tested whether enhanced O_2_ tolerance could enable the FO strain to grow under oxic conditions. Therefore, several pretreatment methods that are typically applied to remove O_2_ from a culture vessel were omitted, namely the addition of reducing agent Na_2_S, autoclaving, and inert gas purging. In the bioreactor, the growth of the wild-type strain was severely impaired under oxic conditions, as expected (Fig. 4a). In contrast, after a prolonged lag phase, the FO strain started to grow and reached a cell density similar to that obtained under anoxic conditions (Fig. 4a). Maximum specific growth rates were quite similar in the two conditions (Table 1). This result is quite surprising, considering that the experimental strain used in this study, *T. onnurineus* NA1, is an obligate anaerobe. To our knowledge, it has never been reported that the overexpression of only one gene caused an obligate anaerobe to grow under oxic conditions. Then we sought to examine what would happen to H_2_ production under oxic conditions. Although H_2_ production was closely correlated with growth on formate under anoxic conditions [10], the maximum H_2_ production rate and maximum specific H_2_ production rate were approximately 90% of those under anoxic conditions (Fig. 4b and Table 1). In comparison with the wild-type strain cultured under anoxic conditions, the FO strain under oxic conditions showed 1.6- to 3.2-fold higher maximum specific growth rate, maximum H_2_ production rate, and maximum specific H_2_ production rate (Table 1).

**Table 1:** Kinetic analysis of the wild-type and FO strains under oxic or anoxic conditions

Kinetic parameter	Wild type		FO^b^	
	Anoxic^a^	Oxic	Anoxic	Oxic
μ_max_ (h^−1^)	0.3	ND	0.60 ± 0.08	0.60 ± 0.02
*r*_max_ (mmol L^−1^ h^−1^)	31.7	ND	118.5 ± 3.9	101.7 ± 16.6
*q*_max_ (mmol g^−1^ h^−1^)	198.2	ND	364.8 ± 8.1	323.3 ± 54.5

*μ*_*max*_ maximum specific growth rate, *r*_*max*_ maximum H_2_ production rate, *q*_*max*_ maximum specific H_2_ production rate, *ND* growth and H_2_ production were not detected

^a^The data on the kinetic parameters of the wild-type strain under anoxic conditions were adapted from a previous study [23]

^b^The values for the FO strain show mean ± standard deviation for three independent cultures in this study

Taken together, the overexpression of *frhAGB* genes endowed *T. onnurineus* NA1 with a substantial increase in tolerance to O_2_. This O_2_-tolerant property is a significant advantage in biotechnological applications because it is not necessary to strictly protect the growth and H_2_ production processes from O_2_. H_2_ production under micro-aerobic conditions would be a cost-effective and time-saving process.

## Discussion

When the *frhAGB* gene cluster was overexpressed in *T. onnurineus* NA1, a recombinant strain (FO) showed better growth than the wild-type strain. The molecular mechanism underlying the enhanced cell growth is not yet understood. Instead, we attempted to exploit the potential of the FO strain using information from whole transcriptomic analysis. The expression of antioxidant-related genes encoding peroxiredoxin and alkyl hydroperoxide reductase subunit c was 2.5- and 4.1-fold up-regulated under the anaerobic conditions, respectively. The transcriptional up-regulation of antioxidant-related genes is usually associated with cellular responses to oxidative stress. However, the sources for oxidative stress are unidentified. Extracellular O_2_ is usually removed by the addition of a reducing agent, autoclaving the medium, and inert gas purging. The absence of O_2_ in our experiment was evidenced by minimal or no change in the transcriptional expression of other antioxidant-related genes, such as thioredoxin peroxidase (TON_0862), rubrerythrin (TON_0864), rubrerythrin-related protein (TON_0873), NAD(P)H rubredoxin oxidoreductase (TON_0865), and thioredoxin reductase (TON_1603), which were identified to be strongly up-regulated in the presence of O_2_ (manuscript forthcoming). We speculated that the FO strain, with two antioxidant-related genes up-regulated, might exhibit changes in O_2_ tolerance. To verify this hypothesis, the O_2_ sensitivities of the FO strain, wild-type strain, and *ΔfrhA* mutant were compared. The expression level of the *frhAGB* gene cluster had a strong influence on oxidative stress defense. In the *ΔfrhA* mutant, however, the transcript levels of genes encoding peroxiredoxin and alkyl hydroperoxide reductase subunit c were similar to those in the wild-type strain [28]. This result implies that there are more genes that contribute to oxidative stress defense in the FO strain.

O_2_ tolerance in obligate anaerobes by means of hydrogenases has been reported previously. In obligate anaerobic bacteria *Desulfovibrio vulgaris* strains, H_2_-consuming periplasmic soluble hydrogenase and *c*-type cytochrome couples were identified to play a role in O_2_ reduction, thereby protecting against oxidative stress [29, 30]. It has been reported that the hydrogenase was up-regulated when *D. vulgaris* Hildenborough was exposed to O_2_ [30]. In another obligate anaerobic bacteria *Geobacter sulfurreducens*, a periplasmic hydrogenase Hya was identified to be necessary for growth after exposure to oxidative stress. While the Hya-deficient strain was more sensitive to the presence of superoxide or hydrogen peroxide, overexpression of Hya enabled the strain to endure oxidative stress better than the wild-type strain. However, the mechanism by which this hydrogenase contributed to the defense against oxidative stress relative to the promotion of antioxidant enzyme activity was not elucidated [31].

In this study, the FO strain was distinct in that it was able to achieve similar growth yields under oxic conditions, where most anaerobes and the wild-type *T. onnurinues* NA1 strain are incapable of growth due to the high O_2_ level, compared with those of anoxic conditions. In addition, the maximum H_2_ production rates and maximum specific H_2_ production rates of the FO strain cultured under both oxic and anoxic conditions were quite similar to the high values for the WTF-156T strain, which had been engineered by adaptive evolution on formate-supplemented medium [23]. This feature can ameliorate the need to tightly maintain the anaerobic environment prior to cultivation in a batchwise or continuous system, where a certain level of O_2_ contamination is inevitable, by operating with the addition of carbon sources or other nutrients, or by maintaining a constant pH using strong acid or bases. Therefore, the ability of the FO strain to tolerate O_2_ would make it even more suitable for industrial applications. Our study implicates that other anaerobic H_2_ producers might be relieved from the strict control of O_2_ during growth through augmentation of the defense against oxidative stress. It would be helpful if we could get high amounts of whole cell biocatalysts and even purified enzymes from them easily by growing those strains under oxic conditions while the activity of enzymes, such as hydrogenases, FHLs, and HDCRs, essential for H_2_ production [12, 14, 16, 17], is untouched.

This study helps to understand the physiological role of the *frhAGB*-encoding hydrogenase, which is distinct from Frh hydrogenases of methanogens and is thus far poorly understood. In our previous report on the *frhA*-deletion mutant, it was shown that the hydrogenase might be associated with the regulation of gene expression in a non-methanogen [28]. For example, *frhA* gene deletion caused up-regulation of the *codh*–*mch*–*mnh3* gene cluster essential for CO-dependent H_2_ production even in the absence of external CO and led to significant increases in cell growth (2.8-fold) and H_2_ production (3.4-fold) [18]. In this study, overexpression of *frhAGB* genes also up-regulated the expression of antioxidant-related genes without exposing them to an oxidizing agent such as O_2_. Furthermore, the FO strain was superior to the wild-type strain with respect to growth under oxic conditions. Regulatory hydrogenases HupUV and HoxBC have been reported to participate in the transcriptional regulation of gene expression [25]. These hydrogenases function as H_2_ sensors in two-component regulatory systems, which consist of a protein histidine kinase and a response regulator [32]. Two-component regulatory systems are distributed widely in bacteria, but only a few are found in archaea [33]. Therefore, if the soluble hydrogenase is playing a regulatory role, its mechanism in *T. onnurineus* NA1 awaits further studies. It is noteworthy that two genes encoding transcriptional regulatory proteins (TON_0836 and TON_1663) were down-regulated by overexpression of *frhAGB* genes. Further studies will be required to address whether these regulators are involved in the regulation of gene expression by the *frhAGB*-encoding hydrogenase. Contributions of other up- or down-regulated genes by overexpression of *frhAGB* genes cannot be ruled out; therefore, this issue requires further investigation.

## Conclusions

In this study, we demonstrated that the overexpression of *frhAGB*-encoding hydrogenase genes significantly enhanced O_2_ tolerance of obligate anaerobe *T. onnurineus* NA1. This engineered strain overcame the inhibitory effects of O_2_ and showed growth and H_2_ production under oxic condition. This study gives an insight into the development of biotechnologically useful anaerobic H_2_ producer and identification of an unknown function of the hydrogenase in *T. onnurineus* NA1.

## Methods

### Strain, medium and culture conditions

The *T. onnurineus* NA1 wild-type (KCTC 10859), FO strain overexpressing *frhAGB* [27], and *frhA*-deletion mutant [28] strains were cultured at 80 °C in modified medium 1 (MM1) with 1% sodium formate (MM1-F) as previously described [10, 34]. In serum vial cultivation, the initial pH of the medium was adjusted to 6.5. In bioreactor cultivations, pH was maintained at 6.1–6.2, and MM1-F medium was modified as previously described [23].

### Analytical methods

Cell growth was measured by the optical density value at 600 nm (OD_600_) using a UV–visible spectrophotometer (BioPhotometer Plus; Eppendorf, Hamburg, Germany). The dry cell weight (DCW) was deduced by the previous determination of the correlation between OD_600_ and DCW values [18]. The H_2_ concentration in the headspace was measured as previously described [10]. The H_2_ production rate in the bioreactor experiment was determined as previously described [18].

### SDS-PAGE and Western blot analysis

Cells were disrupted by sonication in 50 mM Tris–HCl buffer (pH 8.0) containing 100 mM NaCl, 19 mM KCl, 5% glycerol and a protease inhibitor cocktail tablet (Roche Applied Science, Madison, USA) and were analyzed by 12% sodium dodecyl sulfate polyacrylamide gel electrophoresis (SDS-PAGE). Western blots were performed using primary polyclonal antibodies specific for the *frhA*-encoding protein or a large subunit of Mfh2 hydrogenase and were analyzed using a chemiluminescent dye with the Immun-Star horseradish peroxidase chemiluminescent kit (Bio-Rad, Hercules, USA).

### O_2_ sensitivity test

To test O_2_ sensitivity of cells, atmospheric air (21% O_2_, v/v) or pure O_2_ was injected into the headspace of culture vials after inoculation and the optical density of cells grown at 80 °C for 12 h was measured at 600 nm (OD_600_). To cultivate cells in a bioreactor under oxic condition (presence of O_2_ in the headspace), reducing agent Na_2_S was omitted and autoclaving and inert gas purging were not performed.

### Transcriptome analysis

Transcriptomic profiling of the FO strain overexpressing *frhAGB* was carried out using mRNA samples prepared from two independent cultures. Manufacturing of the microarray slides, microarray processing and signal normalization were performed as previously described [10]. The microarray raw data have been deposited in the National Center for Biotechnology Information Gene Expression Omnibus (NCBI GEO) with the accession code GSE88718.

## Additional file

**Additional file 1: Table S1.** Genes identified as up-regulated by overexpression of *frhAGB* genes based on the transcriptome analysis. **Table S2.** Genes identified as down-regulated by overexpression of *frhAGB* genes based on the transcriptome analysis. **Figure S1.** Construction of the FO strain. **Figure S2.** Protein level of Mfh2 in the wild-type and FO strains.
