# Supplementary material for: Biohydrogen production of obligate anaerobic archaeon Thermococcus onnurineus NA1 under oxic conditions via overexpression of frhAGB-encoding hydrogenase genes
Source: Biotechnol Biofuels. 2019 Feb 8;12:24. doi: 10.1186/s13068-019-1365-3 (PMC6367845; doi:10.1186/s13068-019-1365-3)
Supplement: Supplementary file 1 — Additional file 1: Table S1. Genes identified as up-regulated by overexpression of frhAGB genes based on the transcriptome analysis. Table S2. Genes identified as down-regulated by overexpression of frhAGB genes based on the transcriptome analysis. Figure S1. Construction of the FO strain. Figure S2. Protein level of Mfh2 in the wild-type and FO strains. [file 13068_2019_1365_MOESM1_ESM.docx]

**Additional file 1: Table S1.** Genes identified as up-regulated by overexpression of *frhAGB* genes based on the transcriptome analysis. **Table S2.** Genes identified as down-regulated by overexpression of *frhAGB* genes based on the transcriptome analysis. **Figure S1.** Construction of the FO strain. **Figure S2.** Protein level of Mfh2 in the wild-type and FO strains.

**Table S1 Genes identified as up-regulated by overexpression of *frhAGB* genes based on the transcriptome analysis.**

| Locus tag | Annotation | Mean intensity ratio  (log_2_ ± SD)^a^ | Expression ratio  (FO/wild-type)^b^ | *P-*value ^a^ |
| --- | --- | --- | --- | --- |
| TON_0014 | ABC transporter tungsten-binding protein | 1.3 ± 0.1 | 2.4 | 0.00 |
| TON_0015 | Binding-protein-dependent transport systems inner membrane component | 1.2 ± 0.4 | 2.3 | 0.02 |
| TON_0016 | Molybdenum-pterin binding domain | 1.3 ± 0.4 | 2.5 | 0.01 |
| TON_0049 | Subtilisin-like serine protease precursor | 1.4 ± 0.1 | 2.7 | 0.00 |
| TON_0209 | Metallophosphoesterase, calcineurin superfamily | 1.2 ± 0.1 | 2.3 | 0.00 |
| TON_0260 | Cobalt/zinc/cadmium cation efflux pump protein | 1.5 ± 0.2 | 2.9 | 0.00 |
| TON_0656 | Iron(II) transport protein A | 1.4 ± 0.3 | 2.7 | 0.03 |
| TON_0657 | Iron(II) transport protein B | 2.1 ± 0.3 | 4.2 | 0.01 |
| TON_0658 | LexA-related DNA-binding protein | 1.2 ± 0.2 | 2.3 | 0.00 |
| TON_0786 | Peroxiredoxin, bacterioferritin comigratory protein homolog, AhpC/TSA family | 1.3 ± 0.5 | 2.5 | 0.01 |
| TON_0847 | Alkyl hydroperoxide reductase subunit c | 2.0 ± 0.3 | 4.1 | 0.00 |
| TON_0853 | Putative thiamin biosynthesis protein thiI | 1.2 ± 0.1 | 2.3 | 0.00 |
| TON_0854 | Molybdopterin biosynthesis protein moeb | 1.0 ± 0.1 | 2.0 | 0.00 |
| TON_0971 | Secreted protein containing cell-adhesion domains | 2.0 ± 0.6 | 4.1 | 0.01 |
| TON_1110 | Methylmalonyl-CoA mutase, N-terminus of large subunit | 1.1 ± 0.9 | 2.1 | 0.14 |
| TON_1213 | Polysaccharide deacetylase | 1.1 ± 0.3 | 2.2 | 0.00 |
| TON_1274 | Membrane-bound metal-dependent hydrolase | 1.1 ± 0.3 | 2.1 | 0.01 |
| TON_1353 | cmo tungsten-containing aldehyde ferredoxin oxidoreductase cofactor modifying protein | 1.2 ± 0.2 | 2.3 | 0.00 |
| TON_1355 | Subtilisin-like serine protease precursor | 1.3 ± 0.5 | 2.5 | 0.01 |
| TON_1493 | Universal stress protein | 1.1 ± 0.4 | 2.1 | 0.01 |
| TON_1655 | Histone macroH2A-related protein (C-terminus) | 1.4 ± 0.7 | 2.6 | 0.04 |
| TON_1656 | Daunorubicin resistance ATP-binding protein drrA | 1.1 ± 0.6 | 2.2 | 0.06 |
| TON_1877 | Chaperonin β subunit | 1.7 ± 0.7 | 3.2 | 0.08 |
| TON_1950 | 4-hydroxybenzoate octaprenyltransferase | 1.4 ± 0.1 | 2.6 | 0.00 |

^a^These values were calculated as previously described (Lee et al., 2017).

^b^Genes identified as up-regulated transcripts with a expression ratio ≥2 were selected, but to escape bias by overexpression of the *hmg* gene encoding hydroxymethylglutaryl (HMG)-CoA reductase, up-regulated genes (≥2 fold changes) of the Δ*frhA* mutant strain (Lee et al. 2017) were excluded. All four data points of each transcript were used to measure of expression ratio that were determined by dividing each transcripts level of wild-type per FO strain.

**Table S2 Genes identified as down-regulated by overexpression of *frhAGB* genes based on the transcriptome analysis.**

| Locus tag | Annotation | Mean intensity ratio  (log_2_ ± SD)^a^ | Expression ratio  (wild-type/FO)^b^ | *P-*value ^a^ |
| --- | --- | --- | --- | --- |
| TON_0153 | Hypothetical alpha-amylase | 1.4 ± 0.1 | 0.4 | 0.00 |
| TON_0430 | Hypothetical metallophosphoesterase | 1.5 ± 0.2 | 0.4 | 0.00 |
| TON_0495 | Membrane bound hydrogenase, MbxD subunit | 1.0 ± 0.2 | 0.5 | 0.01 |
| TON_0496 | Membrane bound hydrogenase, MbxC subunit | 1.1 ± 0.2 | 0.5 | 0.01 |
| TON_0535 | Cytosolic NiFe-hydrogenase, delta subunit | 1.3 ± 0.2 | 0.4 | 0.00 |
| TON_0675 | tRNA m1G methyltransferase | 1.3 ± 0.4 | 0.4 | 0.00 |
| TON_0798 | Phosphopantothenoylcysteine synthetase/decarboxylase | 1.1 ± 0.3 | 0.5 | 0.00 |
| TON_0836 | Transcriptional regulatory protein | 1.5 ± 0.3 | 0.4 | 0.00 |
| TON_0886 | Hypothetical histidinol-phosphate aminotransferase | 1.0 ± 0.3 | 0.5 | 0.01 |
| TON_0887 | Hypothetical hydrolase | 1.5 ± 0.8 | 0.4 | 0.04 |
| TON_0889 | ATPase | 1.6 ± 1.1 | 0.3 | 0.03 |
| TON_1042 | DEAD/DEAH box RNA helicase | 1.4 ± 0.5 | 0.4 | 0.00 |
| TON_1072 | Chromosome segregation ATPase | 1.2 ± 0.6 | 0.4 | 0.01 |
| TON_1337 | Hypothetical hydrogenase | 1.3 ± 0.5 | 0.4 | 0.03 |
| TON_1344 | Dephospho-CoA kinase | 1.1 ± 0.3 | 0.5 | 0.00 |
| TON_1375 | Ferredoxin:NADP oxidoreductase, beta subunit | 1.1 ± 0.3 | 0.5 | 0.00 |
| TON_1380 | UvrD/REP helicase | 1.0 ± 0.3 | 0.5 | 0.01 |
| TON_1407 | RadB recombinase | 1.5 ± 0.8 | 0.4 | 0.03 |
| TON_1422 | DNA double-strand break repair ATPase | 1.0 ± 1.0 | 0.5 | 0.1 |
| TON_1663 | Transcription regulator | 1.1 ± 1.0 | 0.5 | 0.07 |
| TON_1669 | Oligopeptide transporter | 1.7 ± 0.9 | 0.3 | 0.02 |
| TON_1670 | Cell division GTPase | 1.5 ± 0.5 | 0.4 | 0.01 |
| TON_1703 | Predicted permease | 1.2 ± 1.1 | 0.4 | 0.1 |
| TON_1707 | sat sulfate adenylyltransferase | 1.2 ± 0.4 | 0.4 | 0.02 |
| TON_1772 | glnA glutamine synthetase | 1.4 ± 0.1 | 0.4 | 0.00 |
| TON_1796 | Cyclomaltodextrinase | 1.1 ± 0.1 | 0.5 | 0.00 |
| TON_1857 | Membrane-bound galactosyl-transferase | 1.6 ± 1.2 | 0.3 | 0.07 |
| TON_1858 | Phosphatidylinositol glycantransferase-class A | 1.5 ± 0.8 | 0.4 | 0.05 |
| TON_1859 | β-1,4-galactosyltransferase | 1.6 ± 1.2 | 0.3 | 0.06 |
| TON_1860 | Capsular polysaccharide biosynthesis protein | 2.0 ± 1.6 | 0.2 | 0.1 |
| TON_1861 | Glycosyltransferase | 1.6 ± 0.8 | 0.3 | 0.02 |
| TON_1862 | Glycosyl transferase | 3.0 ± 0.7 | 0.1 | 0.02 |
| TON_1873 | Iron(III) ABC transporter, permease protein | 1.1 ± 0.6 | 0.5 | 0.02 |

^a^These values were calculated as previously described (Lee et al., 2017).

^b^Genes identified as downregulated transcripts with a expression ratio ≥2 were selected, but to escape bias by overexpression of the *hmg* gene encoding hydroxymethylglutaryl (HMG)-CoA reductase, down-regulated genes (≥2 fold changes) of the Δ*frhA* mutant strain (Lee et al. 2017) were excluded. All four data points of each transcript were used to measure of expression ratio that were determined by dividing each transcripts level of FO per wild-type strain.

**
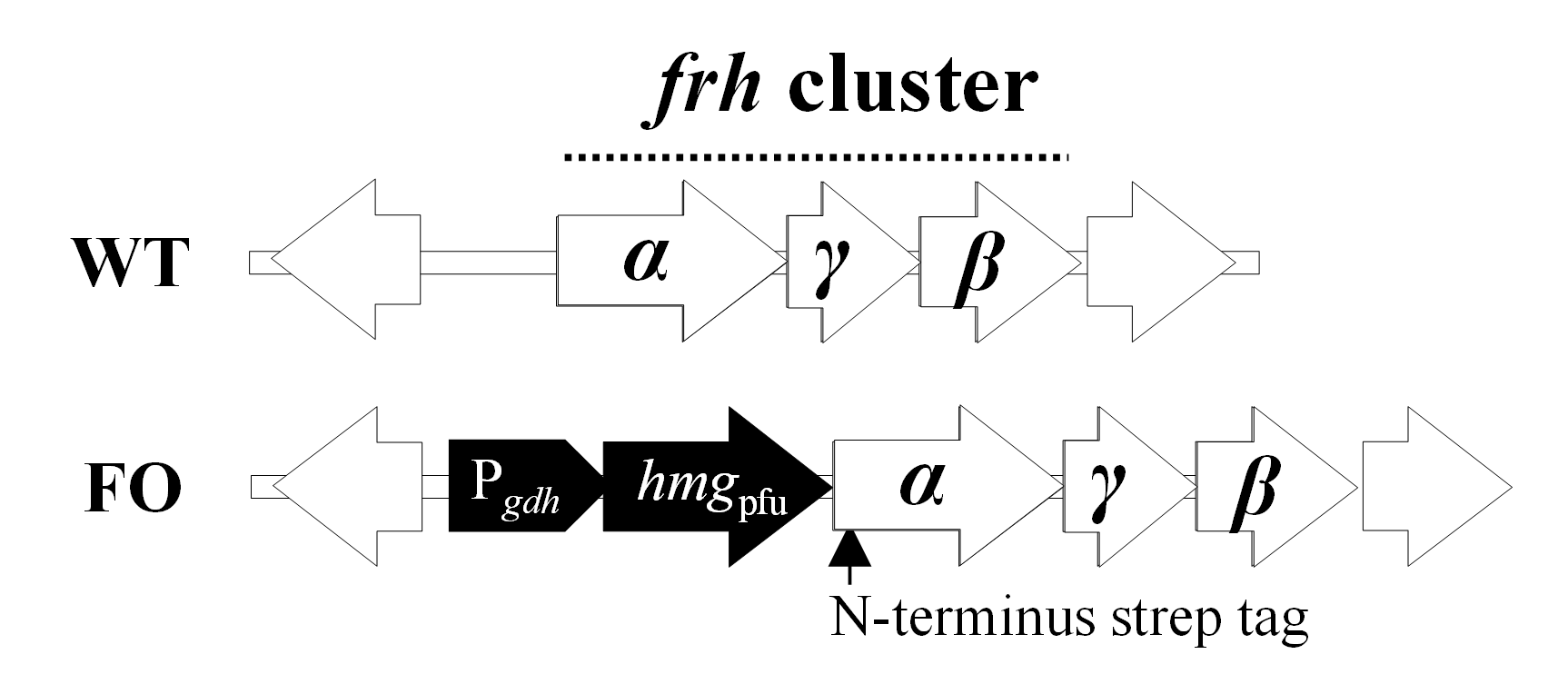
**

**Figure S1.** Construction of FO strain. P*_gdh_* and hmg*_Pfu_* indicate a promoter for the structural gene for glutamate dehydrogenase (gdh) of *Thermococcus kodakarensis* and a 3-hydroxy-3-methylglutaryl coenzyme A (HMG-CoA) reductase gene of *Pyrococcus furiosus*, respectively. WT, wild-type strain; FO, *frhAGB* –overexpressing strain.

**
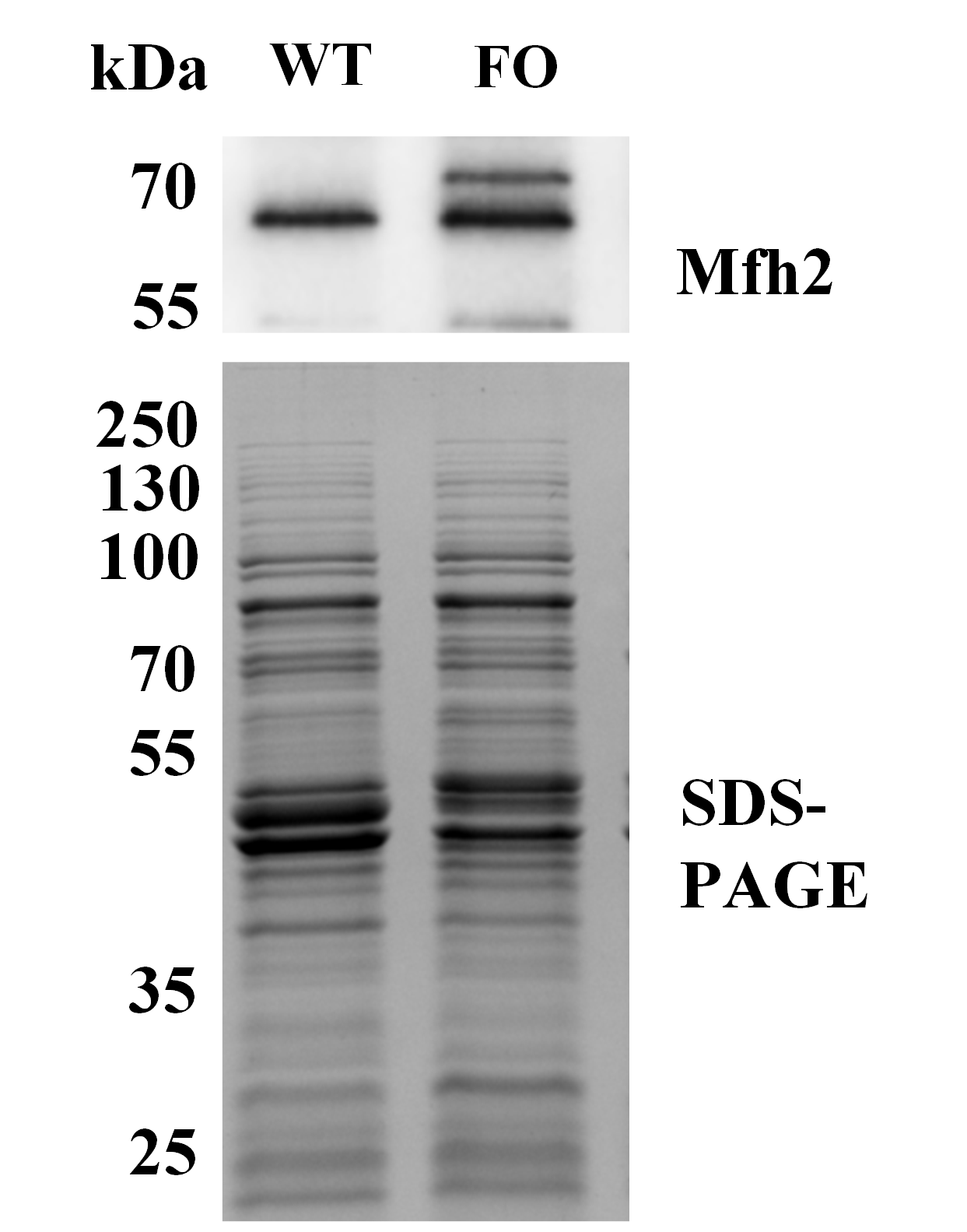
**

**Figure S2.** Protein level of Mfh2 in the wild-type and FO strains. The amounts of large subunit of Mfh2 hydrogenase was monitored by Western blot (upper gel) under formate-supplemented condition. Two Mfh2 bands represent the precursor and the processed form. WT, wild-type; FO, *frhAGB*-overexpressing strain.
